# Pressing needs and recent advances to enhance production of embryos in vitro in cattle

**DOI:** 10.1590/1984-3143-AR2024-0036

**Published:** 2024-08-26

**Authors:** Peter James Hansen

**Affiliations:** 1 D.H. Barron Reproductive and Perinatal Biology Research Program, Department of Animal Sciences, Genetics Institute, University of Florida, Gainesville, FL, United States

**Keywords:** embryo, blastocyst, in vitro production, cattle, recipient, fertility

## Abstract

Embryo transfer in cattle is an increasingly important technique for cattle production. Full attainment of the benefits of the technology will depend on overcoming hurdles to optimal performance using embryos produced in vitro. Given its importance, embryo technology research should become a global research priority for animal reproduction science. Among the goals of that research should be developing methods to increase the proportion of oocytes becoming embryos through optimization of in vitro oocyte maturation and in vitro fertilization, producing an embryo competent to establish and maintain pregnancy after transfer, and increasing recipient fertility through selection, management and pharmacological manipulation. The embryo produced in vitro is susceptible to epigenetic reprogramming and methods should be found to minimize deleterious epigenetic change while altering the developmental program of the resultant calf to increase its health and productivity. There are widening opportunities to rethink the technological basis for much of the current practices for production and transfer of embryos because of explosive advances in fields of bioengineering such as microfluidics, three-dimensional printing of cell culture materials, organoid culture, live-cell imaging, and cryopreservation.

## A call to prioritize embryo technology research

Embryo transfer in cattle has been a reality since December 19, 1950 when Elwyn Willet and colleagues at the American Foundation for the Study of Genetics and the University of Wisconsin produced the first calf (named “Prima”) derived from transfer of an embryo (Willett et al., 1951; Betteridge, 2000). However, the utility of embryo transfer as a tool for genetic improvement was limited until the introduction of the first high density SNP chip for cattle in 2009 (Matukumalli et al., 2009). This chip made it possible to identify genetically-superior females with high reliability. Until then, genetic progress from embryo transfer was hampered by the fact that accuracy of selecting females was poor when compared to the high degree of accuracy conferred by progeny testing of bulls. The utilization of embryo transfer has almost doubled since the introduction of genotyping platforms. The Data Retrieval Committee of the International Embryo Transfer Association reported 794,397 embryos had been transferred in 2008 (Thibier, 2009). The same committee found that the number of transfers reported in 2022 was 1,558,482 (Viana, 2023).

The importance of embryo technologies will continue to rise. Embryo transfer can increase the rate of genetic selection by increasing the intensity of genetic selection on the female side (VanRaden, 2020) and by shortening the generation interval. Genetic selection can be made as early as the blastocyst stage (Agerholm et al., 2023). Somatic cell nuclear cloning and production of gene-edited animals are both technologies that depend on production of embryos in the laboratory and which can contribute to genetic improvement of livestock. A more revolutionary event in the future may be the development of “in vitro breeding” where rounds of genetic selection of embryos produced in vitro are followed by generation of stem cells from those embryos that can be differentiated into sperm cells and oocytes to produce the next generation of embryos for selection (Goszczynski et al., 2023). Embryo transfer can also increase the value of beef calves produced from dairy animals (Crowe et al., 2021) and can be used to improve fertility in heat-stressed and repeat-breeder cows and, eventually, more broadly (Hansen, 2020a). Indeed, the potential impact of embryo technologies on cattle production is so broad that this author has speculated that embryo transfer may eventually rival artificial insemination (AI) as an assisted reproduction technique (Hansen, 2023).

**Figure 1:**
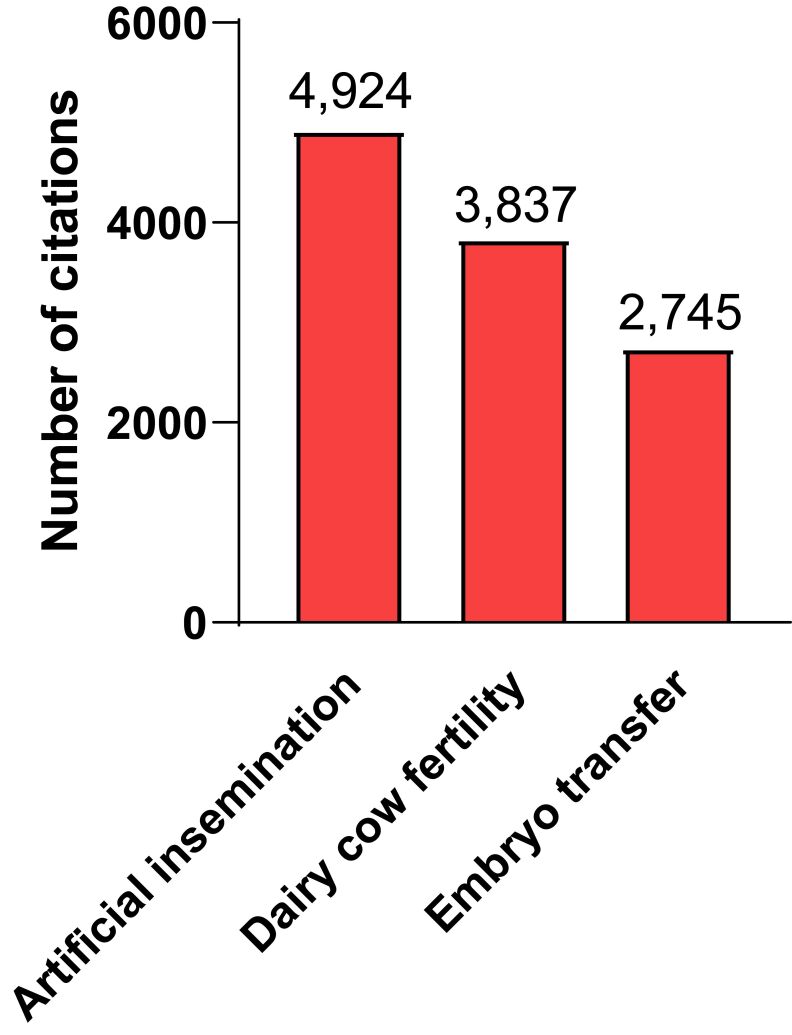
Analysis of PUBMED to assess research activity related to embryo technologies as compared to research focused on artificial insemination and dairy cow fertility. Shown are the number of papers meeting specific search criteria for a search conducted March 7 2024. The search terms related to artificial insemination were “artificial insemination and (cattle or cow or heifer)”. The search terms for dairy cow fertility were “(fertility or infertility or fertile or infertile) and cow and (dairy or Holstein or Jersey)”. The search terms for embryo transfer were “embryo transfer and (cow or cattle or heifer)”.

The scientific developments that have brought embryo technologies to their current standing were achieved with less investment in research than for other important topics in cattle reproduction. Research in bovine reproduction since the 1980s and earlier has been dominated by efforts to develop ovulation synchronization strategies and to improve fertility in the high-producing dairy cow. A search in PUBMED identified 4,924 papers related to AI in cattle, 3,873 papers related to dairy cow fertility and 2,745 papers related to embryo transfer in cattle (Figure 1). Research efforts toward ovulation synchronization research and improving dairy cow fertility have been enormously successful. Timed AI programs are now routinely implemented on many dairy farms in countries where pharmaceutical use is widely available. A host of protocols have been developed that enhance fertility as well as allow appointment breeding (Fricke and Wiltbank, 2022). Dairy cow fertility has been improved because of an assemblage of advances including increased use of ovulation synchronization programs, improved transition cow health programs, enhanced feeding regimens, and other practices. Ovulation synchronization has also made AI in beef cattle more practical than formerly and can result in more calves born earlier in the calving season (Baruselli et al., 2018; Monteiro et al., 2023).

The potential that embryo technologies present for transforming genetic improvement and improving fertility has been limited by suboptimal processes for producing and transferring embryos. The promise offered by the transferrable embryo, coupled with the current obstacles to optimization, means that research to improve embryo technologies should become national priorities for those countries in which cattle production is an important economic activity. Now is the time to increase funding for embryo technologies. In this paper, the goal will be to outline particular areas where research should be focused. The topics are not meant to be inclusive and the coverage of the literature is non-exhaustive. The approach is to highlight some areas that are particularly amenable to improvements or where recent advances show promise.

## Making a better oocyte and zygote

The production of embryos in vitro is very inefficient. The percent of oocytes that are placed into oocyte maturation medium that later become a transferrable embryo is dependent on the culture system but is usually between 20 and 40%. These values are lower than what can be achieved in vivo. For example, the percent of inseminated heifers that was pregnant at day 7 was 54% in a study with beef animals in Ireland (Carter et al., 2008) and 70.9% for dairy heifers in New Zealand (Berg et al., 2022). A total of 56% of lactating cows inseminated at 40-60 days in milk yielded high- or fair-quality embryos at day 5 or 6 after insemination (Denis-Robichaud et al., 2022).

The low yield of transferrable embryos following procedures for in vitro production is not because oocytes fail to mature or become fertilized. Indeed, rates of nuclear maturation and fertilization are high (>70%). Instead, it is because many fertilized embryos fail to develop adequately in culture. They fail largely because of errors in the process of in vitro maturation and fertilization.

**Figure 2:**
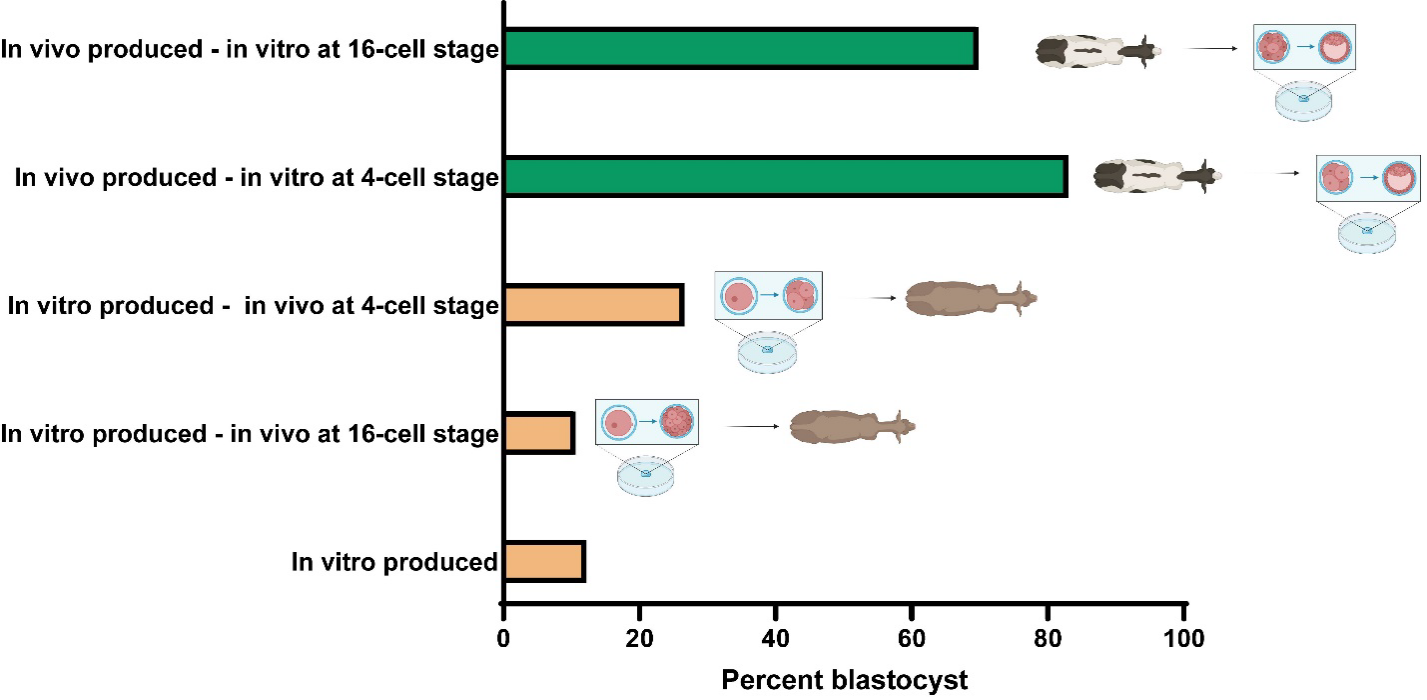
Percent of oocytes becoming blastocysts where embryos were produced in vivo, in vitro or in a combination of both conditions. Data are obtained from Gad et al. (2012).

The conclusion that inadequate conditions for maturation and fertilization are a major cause of poor embryo competence are based on experiments in which measures of embryonic development were made for embryos produced in vivo, in vitro or in a combination of both conditions. Results of one such experiment, by Gad et al. (2012), are shown in Figure 2. The percent of oocytes that became blastocysts following in vitro maturation, fertilization and embryonic development was 12.2%. If in vitro produced embryos were transferred to the uterus at the 16-cell stage, there was no improvement in blastocyst development (10.6%) while transfer to the oviduct at the 4-cell stage caused a slight increase in development (26.6%). In contrast, production of embryos by superovulation, followed by flushing from the animal and subsequent culture at either the 4-cell stage or 16-stage resulted in most oocytes becoming blastocysts (83.0% and 69.8%, respectively). What was most crucial to ensuring blastocyst development was the period of oocyte maturation, fertilization, or development through the 4-cell stage. Findings of two earlier experiments by Rizos et al. (2002) also highlighted the importance of oocyte maturation and fertilization. In the first experiment, the percent of putative zygotes (i.e, oocytes exposed to sperm) becoming blastocysts if maturation, fertilization and embryo development occurred in vitro was 39%. The percent rose to 78% if oocyte maturation alone occurred in vivo. In the second experiment, percent blastocyst was 39% for embryos produced totally in vitro, 58% for embryos from oocytes that were matured in vivo but fertilized and allowed to develop in vitro, and 74% for embryos from oocytes where maturation and fertilization but not development occurred in vivo.

These results indicate that the zygote formed from in vitro maturation and fertilization is already compromised in its ability to proceed to the blastocyst stage. A similar conclusion can be derived from experiments with other species including the human, mouse and rhesus monkey (Hansen, 2020b). There is a compelling need, therefore, to rethink the conditions for oocyte maturation and fertilization in vitro. Perhaps, the most fruitful approaches will be those that try to mimic in vitro the processes for oocyte maturation and fertilization that occur in vivo. There may also be some prospects to improve blastocyst yield by changing conditions for development after fertilization.

### Oocyte maturation

Maturational events in the cumulus-oocyte complex in vivo are driven by luteinizing hormone (LH) and mediators of LH action like amphiregulin, neuroregulin, epiregulin and betacellulin (Strączyńska et al., 2022). Key events include dynamic changes in oocyte concentrations of cGMP and cAMP controlled by loss of gap junctions between cumulus cells and oocytes. Premature loss of oocyte cAMP in vitro is believed to result in premature nuclear maturation and a decoupling of the processes of nuclear and cytoplasmic maturation (Gilchrist and Thompson, 2007). Artificial regulation of oocyte cAMP during in vitro maturation by pharmacological methods has sometimes (but not always) been reported to increase the proportion of oocytes developing to the blastocyst stage (Gilchrist et al., 2016; Leal et al., 2022).

Another approach to improve oocyte maturation in vitro is to add to culture medium specific cell signaling ligands involved in maturation in vivo including follicle stimulating hormone, amphiregulin, insulin-like growth factor 1, estradiol, progesterone, androstenedione, neuroregulin 1 and natriuretic peptide C. Improvements in the characteristics of blastocysts produced have been reported (Soares et al., 2017; Dellaqua et al., 2023) but the percent of oocytes becoming blastocysts is still below what is achieved in vivo. Recently, Zhang et al. (2023) reported that C-X-C motif chemokine ligand 12 acts on oocytes during maturation to increase the percent that become blastocysts following fertilization or parthenogenetic activation.

### Fertilization

The experiment by Rizos et al. (2002) comparing blastocyst development for oocytes fertilized in vivo vs in vitro is indicative that conditions for in vitro fertilization are not optimal for developmental competence of the resulting embryo. Sperm cells vary in ability to produce embryos with high developmental competence (Hendricks and Hansen, 2010; Behnam et al., 2023; Vallet-Buisan et al., 2023; Li et al., 2023; Yaghoobi et al., 2024). Perhaps the probability of fertilization with a defective spermatozoan is greater for in vitro fertilization than for fertilization in vivo. Sperm concentration for in vitro fertilization is usually 1 x 10^6^/mL while estimated numbers of sperm in the oviduct after insemination are in the 10’s of thousands; the number that reach the site of fertilization are likely to be in the hundreds or lower (Hawk, 1987). It is possible that the sperm winnowing process in the reproductive tract (see Miller, 2024) is such that sperm more fit for fertilization and support of embryonic development have greater likelihood to reach the oocyte than less-fit sperm. In mice, for example, passage through the utero-tubal junction depends upon presence of expression of specific genes in the male such as *Adam3* and *Lypd4* (Fujihara et al., 2019) and sperm with fragmented DNA are less likely to transit the utero-tubal junction (Hourcade et al., 2010).

Given the variability in ability of sperm to support embryonic development, one strategy for improving outcomes of in vitro fertilization is to devise new methods for selecting sperm for fertilization. Selection of sperm based on rheotaxis, for example, increased the proportion of cleaved embryos becoming a blastocyst as compared to oocytes from sperm isolated by centrifugation (Yaghoobi et al., 2024). Another strategy is to mimic sperm-oviductal interactions that occur in vivo. Sperm binding to the oviductal epithelium promotes sperm survival (Pollard et al., 1991) and, based on experiments in rabbits and pigs, the oviductal isthmus is important for reducing polyspermy (Mahé et al., 2021). Capacitation of sperm in vivo involves complex changes in the sperm mediated by the oviduct (Mahé et al., 2021; Delgado-Bermúdez et al., 2022). Capacitation of sperm in vitro is caused by heparin (Parrish, 2014) and it may be that this glycosaminoglycan does not completely mimic oviduct-induced changes in sperm function associated with capacitation. Delayed or incomplete capacitation could conceivably result in fertilization with a defective sperm or oocyte aging, which can reduce embryo competence for development (Koyama et al., 2014). Co-culture of sperm, oviductal epithelial cells and oocytes in a system termed “oviduct-on-a-chip” resulted in reduced incidence of polyspermy and parthenogenesis (Ferraz et al., 2017).

### Embryo culture

Expression of large number of genes encoding for receptors for cell-signaling ligands by the early embryo (Sang et al., 2021; Hoorn et al., 2023) is evidence that the preimplantation embryo is in active communication with the mother. Addition of oviductal fluid (Lopera-Vasquez et al., 2017) or several specific growth factors whose gene is expressed in the oviduct or endometrium can increase the percent of in vitro produced embryos developing to the blastocyst stage. Examples of molecules that can increase blastocyst yield include activin A (Trigal et al., 2011; Kannampuzha-Francis et al., 2017; Tríbulo et al., 2018), colony stimulating factor 2 (CSF2; Dobbs et al., 2013), C-X-C motif chemokine ligand 12 (Zhang et al., 2023), hepatoma-derived growth factor (Gómez et al., 2017), insulin-like growth factor 1 (Tríbulo et al., 2018), WNT5A (Jeensuk et al., 2022), and WNT7A (Tríbulo et al., 2018).

Cell-signaling ligands that can enhance embryonic function have been termed embryokines (Hansen et al., 2014). One of the features of actions of embryokines in vitro is that the magnitude of the increase in blastocyst yield is modest. It is likely that these molecules cannot overcome the reduction in developmental competence caused by inadequate conditions for oocyte maturation or fertilization. In addition, other components of culture medium, most notably albumin, can alter growth factor biological activity (Gómez et al., 2017; Jeensuk et al., 2022). It is also possible that embryos themselves secrete specific embryokines so addition of an exogenous source of the molecule might not be impactful. Recently, it was surmised that the reason why bovine embryos are more likely to become blastocysts when cultured in groups than when cultured singly (Donnay et al., 1997) is because of embryonic secretion of L-cathepsin. This proteinase is secreted in higher amounts by embryos classified as excellent or good than for embryos classified as poor (Raes et al., 2023). Moreover, addition of L-cathepsin to embryos cultured individually increased the proportion that became blastocysts (Raes et al., 2023).

## Making a better transferrable embryo

**Figure 3:**
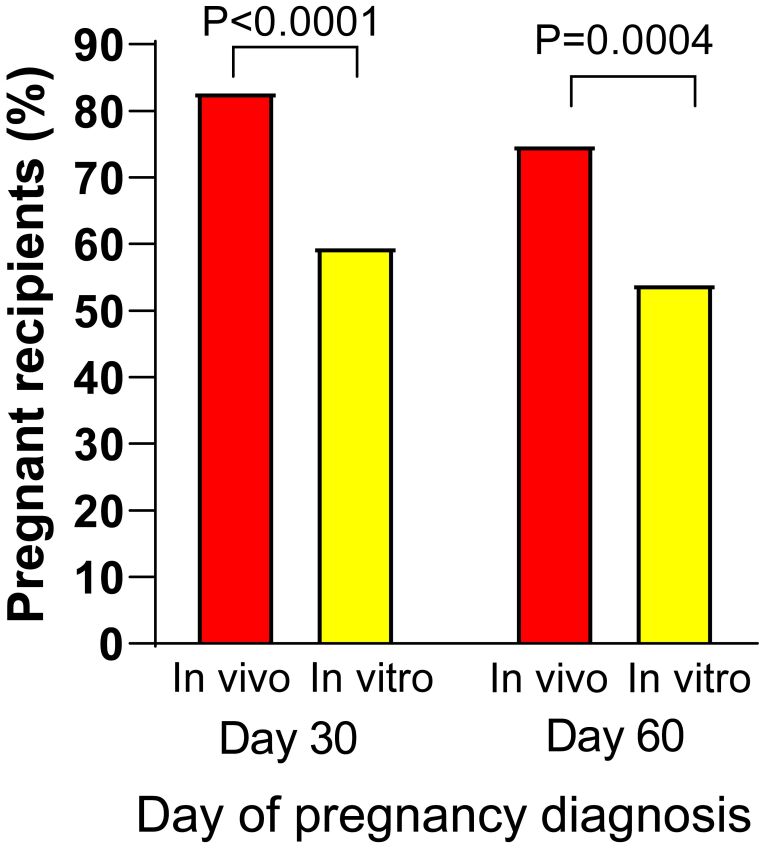
Comparison of pregnancy outcomes for transfer of embryos produced by superovulation (in vivo) or in vitro for beef cows in tropical conditions in Mexico (Pérez-Mora et al., 2020).

The blastocyst produced in vitro differs from its in vivo produced counterpart in many respects including in terms of ultrastructure, lipid content, gene expression, epigenetic modifications, cell numbers, and incidence of chromosomal abnormalities (Hansen, 2023). Not surprisingly, pregnancy rates achieved following transfer of an in vitro produced embryo are often lower than those achieved following transfer of an embryo produced in vivo by superovulation (Ealy et al., 2019). An example of results from one such study comparing pregnancy outcomes for both kind of embryos, that of Pérez-Mora et al. (2020), is shown in Figure 3. In some reports, but not all, pregnancy losses after the initial pregnancy diagnosis were also greater for pregnancies involving embryos produced in vitro than pregnancies established by AI (reviewed by Hansen, 2023; also see Crowe et al., 2024).

It is not known whether reduced competence of the embryo produced in vitro to establish and maintain pregnancy is related to errors associated with oocyte maturation, fertilization, embryo culture or some combination. There are a multitude of experiments describing specific conditions of embryo culture that change blastocyst gene expression, relative or absolute numbers of cells in the trophectoderm (TE) and inner cell mass (ICM), or other characteristics. For example, many of the cell-signaling molecules produced by the endometrium that have embryokine activity can modify allocation of cells of the blastocyst into TE, ICM and hypoblast. Examples of embryokines that have been reported to alter the number of ICM cells in the blastocyst include connective tissue growth factor (Kannampuzha-Francis et al., 2017), C-natriuretic peptide (Sang et al., 2020), interleukin 6 (IL6, Wooldridge et al., 2019; Wooldridge and Ealy, 2021; Seekford et al., 2021), and WNT5A (Jeensuk et al., 2022). Furthermore, Dickopf WNT signaling inhibitor 1 (DKK1), (Denicol et al., 2014), fibroblast growth factor 2 (Yang et al., 2011), and IL6 (Wooldridge and Ealy, 2021) can promote differentiation of hypoblast cells from ICM. Also, DKK1 can increase number of TE cells (Denicol et al., 2014; Amaral et al., 2022a). It is unclear, however, whether any of these embryokines can improve competence of embryos to establish pregnancy. Early experiments indicated that treatment of embryos with CSF2 from day 5 to 7 of development increased pregnancy success after transfer to recipients (Loureiro et al., 2009; Denicol et al., 2014) but a recent meta-analysis of all embryo transfer experiments with CSF2 failed to support the idea that CSF2 increases embryo survival (Hansen et al., 2024). Embryo transfer experiments with DKK1 have also yielded mixed results (Denicol et al., 2014; Amaral et al., 2022b). Pregnancy rates at day 70 for cows receiving an embryo treated with IL6 were numerically but non-significantly higher than for cows receiving control embryos (12/28 vs 11/43) and fetal size for IL6 embryos was more similar to that of fetuses derived by AI than for fetal size of control embryos (Seekford et al., 2021).

One difficulty in understanding determinants of embryo competence for pregnancy is that most embryo transfer experiments designed to test improvements in embryo survival are underpowered. Adequately powered experiments in which pregnancy outcome is the endpoint requires hundreds of observations per treatment. This size of experiment requires resources beyond the reach of most academic laboratories. Commercial laboratories can do these kinds of experiments but it is often not practical to modify a functioning system of embryo production for experimental purposes.

One alternative approach is to identify markers of embryo competence that can be used to rapidly screen potential treatments for improving embryo competence for pregnancy establishment. The most promising treatments could then be tested for efficacy in a large-scale embryo transfer experiment. Recently, Rabaglino et al. (2023) has used machine learning to analyze a variety of datasets on gene expression in bovine embryos including those from demi-blastocysts in which one half of the blastocyst was transferred and one-half was subjected to RNA sequencing. A total of eight genes were identified whose expression together predicted with high accuracy whether pregnancy would result after transfer. Screening of the transcript abundance of these genes could provide insights into the conditions for producing an embryo competent to establish pregnancy. There are also likely to be metabolic signatures of an embryo competent to establish pregnancy as indicated by analysis of spent culture medium of blastocysts that did or did not establish pregnancy after transfer (Oliveira Fernandes et al., 2023).

Another potential method for distinguishing between embryos that are competent or non-competent to establish pregnancy is the use of morphokinetic analysis of embryonic development. Incubators with built-in microscopes and cameras are now available that allow time-lapse imaging of individual embryos as they advance in development (Magata, 2023). Timing of blastocyst formation and specific morphological characteristics of the blastocyst have been found predictive of ability of the embryo to remain viable in culture after day 7.5 (Huayhua et al., 2023). Future experiments to identify morphokinetic determinants of embryonic survival after transfer to recipients could result in a useful tool for increasing embryo competence for pregnancy establishment.

One cause of pregnancy failure in embryos produced in vitro are chromosomal abnormalities. The proportion of embryos with errors in chromosomal segregation is higher for those produced in vitro than for those produced in vivo (Viuff et al., 1999; Tšuiko et al., 2017). Some of these embryos are probably discarded before transfer – development is slowest for haploid and polypoid embryos, intermediate for aneuploid embryos and fastest in embryos classified as diploid or mixoploid (Kawarsky et al., 1996). Incidence of chromosomal abnormalities is also inversely related to blastocyst quality grade and stage of development (Tutt et al., 2021). Recently, it was demonstrated that embryo biopsies used to estimate genetic merit of embryos can also be analyzed to identify chromosomal abnormalities (Bouwman and Mullaart, 2023). Thus, embryos can be simultaneously screened for both genetic merit and chromosomal abnormalities.

## Making a better calf epigenetically

The preimplantation embryo undergoes extensive epigenetic modifications involving removal of epigenetic marks inherited from the egg and sperm followed by acquisition of epigenetic modifications required for differentiation (Zhu et al., 2021). This process can be modified by culture of the embryo (Niemann et al., 2010; Urrego et al., 2017; Canovas et al., 2021; Ming et al., 2021). Differences in DNA methylation between calves produced from embryos produced in vitro vs in vivo also exist at birth (Rabaglino et al., 2022). Some of these neonatal differences could represent conserved changes in the epigenome that occurred in the embryo and others could represent changes in methylation downstream from cellular changes in the blastocyst. At least some changes in DNA methylation occurring in the embryo are not retained in the adult (Vargas et al., 2023).

In any case, there is some evidence that in vitro production can result in changes in function of the animal at maturity. In one study, it was reported that cows derived from in vitro fertilization with reverse-sorted semen had reduced milk production compared to cows derived from embryos produced by AI, superovulation, or in vitro fertilization with conventional semen (Siqueira et al., 2017). In that same study, calf mortality was highest for the reverse sex-sorted group. In contrast, there was no difference in milk yield between cows produced by AI, superovulation or in vitro production (Lafontaine et al., 2023). There was, however, a slight increase in interval from first service to conception in the cows derived by in vitro production. It should be noted that characteristics of the epigenome of the blastocyst produced in vitro depend upon conditions of culture (Canovas et al., 2021; Clare et al., 2021; Tutt et al., 2023). Thus, consequences of in vitro production of embryos could depend on the specific methodology utilized.

**Figure 4:**
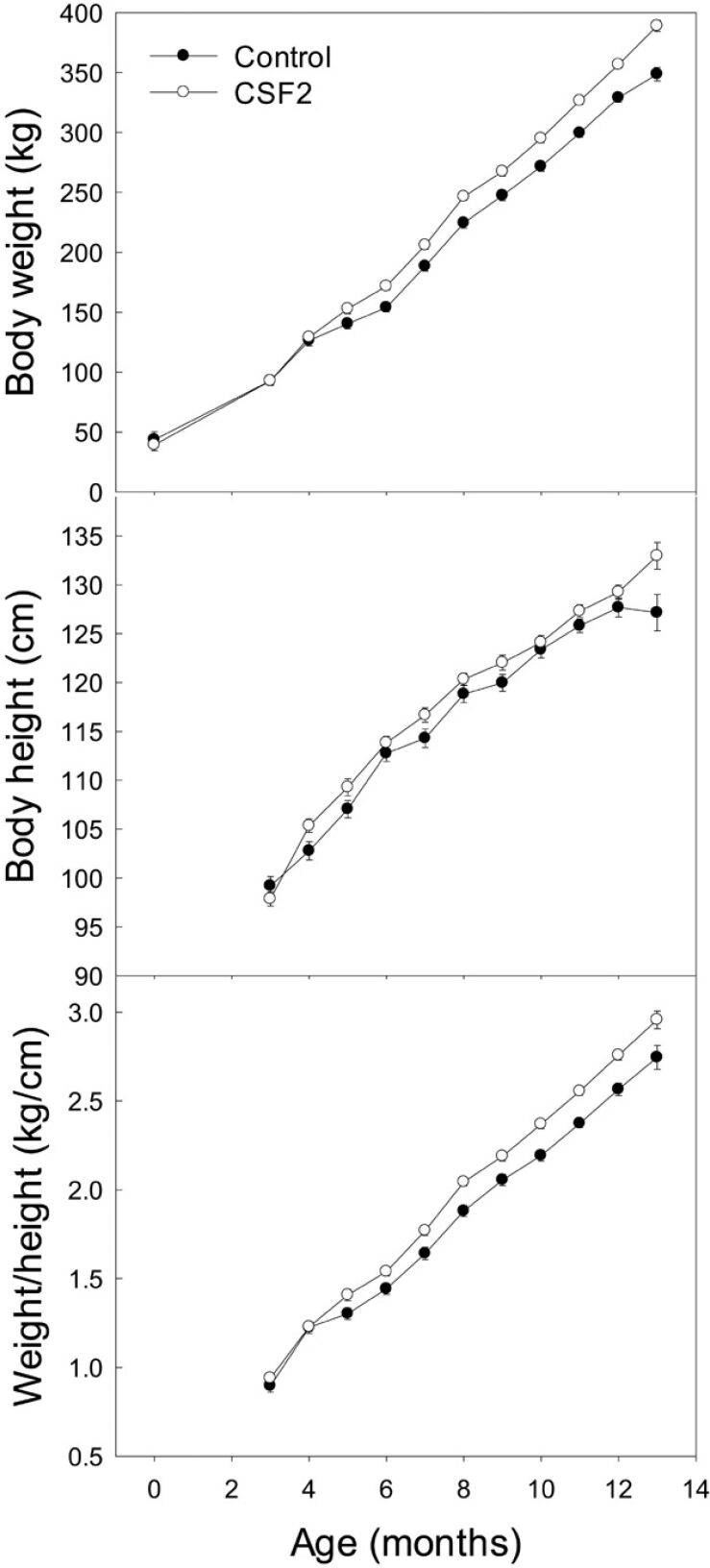
Programming of postnatal growth by exposure of preimplantation embryos to colony stimulating factor 2 (CSF2) from day 5 to 7 of culture. Data are body weights of calves derived from control embryos or embryos cultured with CSF2. Data are least-squares means ± standard error (error bars are not visible when errors were smaller than the symbol). The interaction between treatment and month of age affected weight (*P*< 0.001) and weight-to-height ratio (*P*= 0.0089). The figure is from Kannampuzha-Francis et al. (2015) and is reproduced with permission from *Molecular Reproduction and Development*.

The developmental program of the preimplantation embryo is malleable and a variety of changes in the environment of the mother or embryo can program development to affect postnatal phenotype (Hansen et al., 2016; Hansen, 2020b). As a result, it might be possible to alter culture conditions of the preimplantation embryo to enhance postnatal phenotype. Two embryokines and one nutrient have been reported to do so. As shown in Figure 4, CSF2 has been reported to increase postnatal growth in Holstein heifers (Kannampuzha-Francis et al., 2015). No such effect was seen in Brahman calves (Estrada-Cortés et al., 2021a), which unlike Holsteins, were suckled by their dams. In the latter study, though, CSF2 treatment affected deposition of subcutaneous fat. Calves derived from DKK1-treated embryos were larger at birth than calves derived from control embryos but grew slower than controls thereafter (Amaral et al., 2022a). Culture of in vitro produced embryos with the micronutrient choline has been reported to change DNA methylation in the Brahman calf and increase weaning and slaughter weight (Estrada-Cortés et al., 2021b; Haimon et al., 2024).

**Figure 5:**
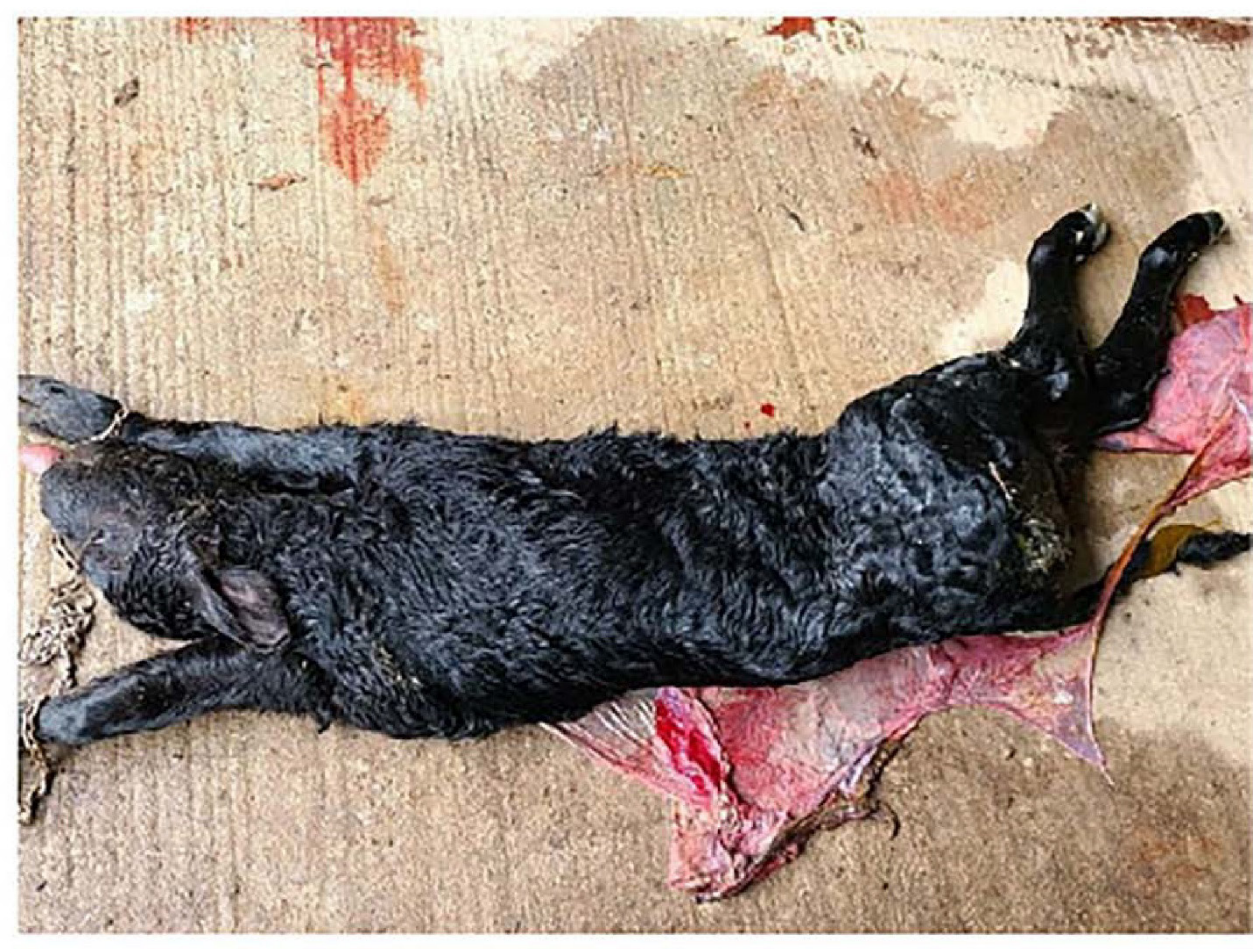
Example of a stillborn bull calf with large offspring syndrome. Body weight was 59 kg and macroglossia was present. The figure is from Amaral et al. (2022a) and is reproduced with permission from *Biology of Reproduction*.

The most deleterious example of disordered development associated with in vitro production is large offspring syndrome (LOS) or, as it is more accurately termed, abnormal offspring syndrome (Farin et al., 2010). The most obvious representation of the syndrome is the extremely large size at birth of affected calves (Figure 5). Other developmental defects including umbilical hernia, organomegaly, abdominal wall defects, and changes in DNA methylation are associated with the condition (Nava-Trujillo and Rivera, 2023). Once thought to be exclusively associated with in vitro production of embryos and somatic cell nuclear transfer, it is now clear that LOS can occur with natural mating or AI (Nava-Trujillo and Rivera, 2023). There are no reliable estimates on the incidence of the syndrome - even the definition of LOS varies among investigators. In our laboratory, the frequency of calved produced by in vitro fertilization that have LOS (defined as very large calves that are born dead or die soon after birth) has been about 5%. The fact that the frequency is so low means it will be difficult to perform experiments to identify culture conditions that minimize the incidence. The best solution will be to identify markers of the syndrome in the embryo or pregnant cow and terminate pregnancies with a high probability of development of a LOS calf. Efforts to identify such markers are underway (Rivera et al., 2022).

## Increasing receptivity of the recipient

Pregnancy success after embryo transfer depends not only on the competence of the embryo to establish pregnancy but also on the ability of the recipient to support pregnancy. Using data from experiments in which two potential pregnancies per female were established, McMillan (1998) estimated that only about 50% of females were capable to maintaining a pregnancy vs 70% of embryos produced in vivo and 60% of embryos produced in vitro. Moreover, experiments in which females underwent repeated rounds of embryo transfer make it clear that some cows are inherently and repeatedly fertile while others are inherently subfertile (Geary et al., 2016). It is to be expected that pregnancy success after embryo transfer will be increased if methods are developed to 1) either identify inherently-fertile cows or 2) to manage cows so that the proportion of recipients that are fertile (i.e., receptive) is increased.

There are physiological characteristics that distinguish cows on their basis for supporting pregnancy. Cows that display estrus in fixed-time embryo transfer procedures have higher pregnancy rates than cows that do not (Cedeño et al., 2020). Blood flow to the corpus luteum at the time of transfer is also positively related to pregnancy outcomes (Kanazawa et al., 2016; Pugliesi et al., 2019; Santos et al., 2023). Other markers of receptivity include expression of specific mRNA (Ponsuksili et al., 2012) and miRNA in the endometrium (Ponsuksili et al., 2014).

Fixed-time embryo transfer is widely used as a management tool for recipients; work continues on optimizing the hormonal treatments employed for this procedure (Pereira et al., 2013; Bonacker et al., 2020). Efforts to improve pregnancy outcomes by modifying the endocrine environment around the time of embryo transfer has yielded mixed results, with reports of both positive effects and lack of effectiveness. This has been the case for treatment with progesterone, GnRH and human chorionic gonadotropin (Block et al., 2003; Monteiro et al., 2015; Niles et al., 2019; García-Guerra et al., 2020; El-Azzi et al., 2023; Chen et al., 2023; Laurindo et al., 2024). A treatment with a more consistent benefit on pregnancy outcomes is administration of flunixin meglumine or other anti-inflammatories at the time of embryo transfer (Besbaci et al., 2021; Barnes et al., 2023). These treatments reduce inflammation associated with the process of transfer itself since they are particularly effective at increasing pregnancy rate in cows in which it was difficult to pass the embryo transfer pipette through the cervix (Besbaci et al., 2021).

## Improving embryo freezability

In 2022, 44% of the global total of recorded transfers of embryos produced in vitro involved a cryopreserved embryo (Viana, 2023) even though pregnancy success after transfer of a cryopreserved embryo remains lower than for transfer of a fresh embryo (Hansen 2020a, 2023). Enhancements in the conditions for production of embryos could conceivably reduce the difference in pregnancy rates between cryopreserved and fresh embryos. So too could enhancements in techniques for cryopreservation. The ideal cryopreservation system would be one where an embryo could be transferred directly without the need for washing and repackaging because transfers could be performed in locations where trained technicians were not available. Gómez et al. (2020) has reported that freezing in an ethylene-glycol based cryoprotectant solution with polyvinyl alcohol instead of bovine serum albumin yielded pregnancy rates similar to those with transfer of fresh embryos [40/80 (50% vs 30/58 (52%). Similarly, Oliveira et al. (2020) reported an in-straw warming protocol for direct transfer of vitrified embryos involving transfer of an embryo from an open vitrification device into an embryo transfer straw held upright and containing liquid columns of 0.15 M sucrose and phosphate-buffered saline. Ten of 25 recipients (40%) receiving such warmed embryos became pregnant as compared to a rate of 43% for recipients receiving fresh embryos. Other experiments have been performed to determine whether addition of various biologically-active molecules to embryos around the time of cryopreservation improves embryonic survival. Examples in which some improvement in survival was reported include antifreeze glycoprotein 8 (Liang et al., 2017), ascorbate (Carrascal-Triana et al., 2022), and an inhibitor of Rho-associated coiled-coil containing kinase (Abdelhady et al., 2023). All of these approaches for enhancing outcomes of embryo cryopreservation are promising but it will be important to perform large-scale embryo transfer experiments to confirm efficacy.

## Designing better tools for embryo production and transfer

There are widening opportunities to rethink the technological basis for much of the current practices for production and transfer of embryos. There have been explosive advances in fields of bioengineering such as microfluidics, three-dimensional printing of cell culture materials, organoid culture, live-cell imaging and cryopreservation. Moreover, artificial intelligence will certainly have a role in embryo technologies. Examples of emerging technologies include microfluidics (Suh et al., 2006; Ferraz and Ferronato, 2023; Alkan et al., 2023), artificial tissues (Gargus et al., 2020), three-dimensional culture (Miles et al., 2017; Ferraz and Ferronato, 2023), non-invasive assessment of cellular function (Sciorio et al., 2022), time-lapse imaging (Magata, 2023) and new advances in cryopreservation (Pomeroy et al., 2022). Even a technique as central to the field as transcervical embryo transfer could be re-engineered to avoid possible damage to the reproductive tract (see Hansen, 2020a for discussion). Could not an automated, autonomous device be designed that could traverse the cervix with minimal physical damage to tissues and without the need for technicians with specialized training?
